# Remedies

**Published:** 1890-02

**Authors:** 


### "REMEDIES."

This is the way a doctor writes about drugs :—As for treatment, I have just been looking over Lauder Brunton, Aitken, Hooper, and some others of the same class. Certainly I had to study them in the days gone by, but just now, for the sake of my fellow men, I have been giving them special attention. Dear me! how can miserable disease live in front of so terrible an array of terrible remedies! What canst thou not have, poor man! Dry cupping, hot poultices, muriate, carbonate, and chlorate of ammonia, aconite, tartar emetic, foxglove, ipecacuanha, paregoric, opium, and its preparations, chloral hydrate, belladonna, colchicum, copaiba, croton oil, quinine, turpentine, lead, jalap, lobelia, mercury, eucalyptus, nitric acid, alum, pilocarpine, turpentine, calabar bean, zinc, potash, thorn apple, strychnia, arsenic, hemlock, petroleum, Indian hemp, and other drugs equally glorious—all are ready to woo the serpent of thy life to rest, or kill thee in the attempt. Truly, I would as soon face a regiment of the Old Guard of France as the formidable drugs mentioned. Never mind, it's an ill wind that blows nobody good, especially registrars of births and deaths.

